# Supplementary material for: Efficacy and safety of ultrasound-guided radiofrequency ablation combined with transhepatic artery embolization chemotherapy for hepatocellular carcinoma: A meta-analysis
Source: PLoS One. 2024 Sep 6;19(9):e0305965. doi: 10.1371/journal.pone.0305965 (PMC11379279; doi:10.1371/journal.pone.0305965)
Supplement: S1 Table — (DOCX) [file pone.0305965.s002.docx]

Table S1

((("Carcinoma, Hepatocellular"[Mesh]) OR (((((((((((((((((((Carcinoma, Hepatocellular[Title/Abstract]) OR (Carcinomas, Hepatocellular[Title/Abstract])) OR (Hepatocellular Carcinomas[Title/Abstract])) OR (Liver Cell Carcinoma, Adult[Title/Abstract])) OR (Liver Cancer, Adult[Title/Abstract])) OR (Adult Liver Cancer[Title/Abstract])) OR (Adult Liver Cancers[Title/Abstract])) OR (Cancer, Adult Liver[Title/Abstract])) OR (Cancers, Adult Liver[Title/Abstract])) OR (Liver Cancers, Adult[Title/Abstract])) OR (Liver Cell Carcinoma[Title/Abstract])) OR (Carcinoma, Liver Cell[Title/Abstract])) OR (Carcinomas, Liver Cell[Title/Abstract])) OR (Cell Carcinoma, Liver[Title/Abstract])) OR (Cell Carcinomas, Liver[Title/Abstract])) OR (Liver Cell Carcinomas[Title/Abstract])) OR (Hepatocellular Carcinoma[Title/Abstract])) OR (Hepatoma[Title/Abstract])) OR (Hepatomas[Title/Abstract]))) AND (("Radiofrequency Ablation"[Mesh]) OR ((((((Radiofrequency Ablation[Title/Abstract]) OR (Ablation, Radiofrequency[Title/Abstract])) OR (Radio Frequency Ablation[Title/Abstract])) OR (Ablation, Radio Frequency[Title/Abstract])) OR (Radio-Frequency Ablation[Title/Abstract])) OR (Ablation, Radio-Frequency[Title/Abstract])))) AND (((transcatheter arterial chemoembolization[Title/Abstract]) OR (TACE[Title/Abstract])) OR (Transarterial Chemoembolization[Title/Abstract]))
