# Supplementary material for: Efficacy and safety of ultrasound-guided radiofrequency ablation combined with transhepatic artery embolization chemotherapy for hepatocellular carcinoma: A meta-analysis
Source: PLoS One. 2024 Sep 6;19(9):e0305965. doi: 10.1371/journal.pone.0305965 (PMC11379279; doi:10.1371/journal.pone.0305965)
Supplement: Attachment — Submitted filename: Response to Reviewers.docx [file pone.0305965.s004.docx]

Dear editor and dear reviewers

Re Manuscript ID (PONE-D-24-11718) entitled " Efficacy and safety of ultrasound-guided radiofrequency ablation combined with transhepatic artery embolization chemotherapy for hepatocellular carcinoma: a meta-analysis". Thank you for your letter and for the reviewer’s comments. Those comments are all valuable and very helpful for revising and improving our paper, as well as the important guiding significant to our research. We have studies comments carefully and have made correction which we hope meet with approval. Revised portion are marked in red in the paper. The main correction in the paper and responds to the reviewer’s are as flowing:

Reviewer #1: Dear authors, thanks for this great, interresting and important work.

- The methodology is good , but I think, it may need more details. Like:

1. How combined TACE and RFA were done (which one was the first modality and what was the time interval between them). And what is the type of chemotherapy in each TACE study.

Responds: Many thanks to the reviewers for their valuable comments, through our careful reading of the articles, we found that most of the included articles performed TACE followed by radiofrequency ablation, but the intervals between the two interventions were not the same in each article, and we added the method of radiofrequency ablation used in each article in Table 1 Literature Characterization Table.

2. Which staging system you used for HCC (BCLC or WHO) and the comparison of staging between each groups (combined TACE and RFA group vs TACE only group).

Responds: Many thanks to the reviewers for their valuable comments, most of the studies we included fall under WHO staging. And some of the studies were not grouped according to interventions for staging, but more directly gave me the staging for all the included population.

3. You didn't mention anything about previous treated patients (were they excluded?)

- I think, it would be good if you made a hint about cost benefit ratio (if possible).

Responds: Many thanks to the reviewers for their questions, which will be a great enhancement to our article, and indeed people who have previously received other treatments will be excluded by us, which we have added to the exclusion criteria.

At the end, it is very benificial work and i am looking forward seeing your reply.

Responds: We strongly agree with the reviewers' comments, but the included articles provided less information for us to cost-effect analyze, but we will dedicate a future study to this cost-effect analysis.

Reviewer #2: The language in submitted article has many errors and repeated sentences carrying the same meaning. Also there are many typographical l errors that should be corrected at revision.

I can not understand the dead line ( a search deadline of 14 March 1, 2024) please clarify

Responds: We are very sorry that we didn't explain it clearly, it means that our retrieval date is March 14, 2024.

Definition of cirrhosis is not correct (cirrhosis (chronic liver damage caused by chronic liver damage caused by fibrosis) it should include regenerating nodules to differentiate cirrhosis from fibrosis.

Responds: Many thanks to the reviewers for pointing out the problems, which we have revised.

Subtitles should be clear and obvious in different size

Responds: Many thanks to the reviewers for pointing out the problems, which we have revised.
